# Patterns of active and passive smoking, and associated factors, in the South-east Anatolian Project (SEAP) region in Turkey

**DOI:** 10.1186/1471-2458-6-15

**Published:** 2006-01-25

**Authors:** Ali I Bozkurt, Saime Şahinöz, Birgül Özçırpıcı, Servet Özgür, Turgut Şahinöz, Hamit Acemoğlu, Günay Saka, Ali Ceylan, Yılmaz Palanci, Ersen İlçin, Feridun Akkafa

**Affiliations:** 1 Department of Public Health, Faculty of Medicine, Pamukkale University, Denizli, Turkey; 2 Department of Public Health, Faculty of Medicine, Gaziantep University, Gaziantep, Turkey; 3 Department of Communicable Diseases, Medical Directorate of Gaziantep Province, Gaziantep, Turkey; 4 Department of Public Health, Faculty of Medicine, Dicle University, Diyarbakır, Turkey; 5 Department of Medical Biology, Faculty of Medicine, Harran University, Sanlıurfa, Turkey

## Abstract

**Background:**

Smoking is an important health threat in Turkey. This study aimed to determine the frequency of and main factors associated with smoking in persons of 15 years and over, and the frequency of passive smoking in homes in the South-east Anatolian Project (SEAP) Region in Turkey.

**Methods:**

A cross sectional design was employed. The sample waschosen by the State Institute of Statistics using a stratified cluster probability sampling method. 1126 houses representing the SEAP Region were visited.

Questionnaires about tobacco smoking and related factors were applied to 2166 women and 1906 men (of 15 years old and above) in their homes. Face-to-face interview methods were employed. Participants were classified as current, ex, and non-smokers. The presence of a regular daily smoker in a house was used as an indication of passive smoking. The chi-square andlogistic regressionanalysis methods were used for the statistical analysis.

**Results:**

The prevalence of smoking, in those of 15 years and over, was 11.8% in women and 49.7% in men. The prevalence of current smokers was higher in urban (34.5 %) than in rural (22.8 %) regions. The mean of total cigarette consumption was 6.5 packs/year in women and 17.9 packs/year in men. There was at least one current smoker in 70.1% of the houses.

**Conclusion:**

Smoking is a serious problem in the South-eastern Anatolian Region. Male gender, middle age, a high level of education and urban residency were most strongly associated with smoking.

## Background

Smoking is an important health threat worldwide. 1.1 billion people smoke globally and 4.5 million people die annually because of smoking. Tobacco is responsible for an average loss of 20 years of life expectancy [1-3]. The economic cost of cigarettes and their consequences on health are significant. The negative effects of smoking on health applies not only to smokers themselves; passive smoking can cause serious health problems to non-smokers as well [4-7]. It has been reported that passive smoking increases the risk of lung cancer and the risk of acute coronary heart disease [8].

Smoking is one of the targets of the World Health Organisation (WHO) policy of 'Health for all in the 21^st ^century'. One of the targets is to reduce the proportion of smokers in the 15 and over age group to lower than 20%. Also, preventing smoking in under-15s in all countries is another aim [3].

Turkey has a population of 70 million and 17 million of these are smokers. Amongst adults of 15 years and over, the percentage of smokers is 44%. An estimated 100 thousand die annually due to smoking. Cardiovascular disease and cancer are the primary and secondary causes of mortality in Turkey, smoking is associated with both [9]. 27% of cases of cancer in Turkey are due to smoking, with smoking leading to 25000 cases of lung cancer annually [10,11].

The SEAP region covers nine provinces and the total population here is more than 6 million. This region includes people of several ethnic backgrounds and it is the poorest region in Turkey. Also the educational level of the population in this region is very low. The prevalence of active and passive smoking and factors related to smoking in the region was investigated in this study.

## Methods

The 'Public Health Project of SEAP' was conducted by a consortium comprising the Turkish Parasitology Association, Gaziantep University, Dicle University (in the province of Diyarbakır) and Harran University (in the province of Şanlıurfa). Permissions from authorities have been obtained (The SEAP Regional Development Management; B.0.2.GAP.0.SPK/19-2773). During the project in 2001 and 2002, data was collected about public health issues and problems of the SEAP region. In the present study, the data collected abouttobacco smoking, passive smoking and factors related to smoking were evaluated.

The total population of the nine provinces in the region was 6.128.973. In order to investigate the public health problems of such a large population, an optimumsample size representing both rural and urban areas of the region was determined to be 6900 (*d *= 0.03, *p *= 0.04, *α *= 0.01). This number (6900) was divided into households (approximately six people live in each house in this region) and the number of houses which would need to be sampled was found to be 1150. After stratifying with regards to residency, the State Institute of Statistics chose houses randomly using an address list by a sampling method proportional to size.

Questionnaires wereprepared by the academic staff in the public health departments of medical faculties in the two universities (Gaziantep and Dicle Universities). The first questionnaire aimed to collect data about socio-demographics and health behaviour of the participants aged 15 and over. The second questionnaire concerned the participant's house and living conditions. Before the study was carried out, these questionnaires were applied to households not in the study sample as a pilot study and then checked.

Teams for eachprovince were formed. The teams consisted of health workers fluent in local languages. Training on the application of the questionnaires was given to each team by the principal investigators to avoid biases and errors related to face-to-face interviews. Several pilot studies were run in the field before the actual study.

Interviewers and a public health specialist went to the selected houses and interviewed each member of the household. Verbal consents from volunteers were obtained after providing necessary information and making explanations about the study. Also verbal consents for minors were obtained from his/her parent or guardian. During face-to-face interviews, in order to increase participation, gender matches between the participants and the interviewers were made.

Although everyone in a selected household was included in the general study, questions about tobacco smokingand related factors were asked only to persons aged 15 or over. If a person was still unavailable after two visits to their house, information on them was obtained from his/her proxy. Data about age, education, employment, ethnic origin, marital status, cigarette use, duration of smoking, amount of cigarettes smoked, and the existence of some respiratory tract symptoms such as haemoptysis, cough, and sputum were obtained. Smoking status was classified as non-smoker, current, or ex-smoker (a cessation of smoking). Current smokers were separated into daily and occasional smokers. In order to indicate total tobacco consumption of the participants, a number of packs smoked/year estimation was used [12].

Data about the features of the house andthe presence (or lack of) passive smoking were obtained by the house questionnaire. The presence of a regular daily smoker in a house was used as an indication of passive smoking.

The data was evaluated on computer using the SPSS 5.0 and Excel programmes. *Chi-square *and *logistic regression analysis *were used for the statistical analysis. Gender, age, education, ethnicity, type of residence, marital and employment status were included in logistic regression analysis as covariates.

## Results

Data was collected from 1126 of the 1150 houses (97.9%). 439 of the houses were in rural areas and 687 of them were in urban areas. A total of 7609 people were living in these houses and 4072 of them were 15 years old and above.

There was at least one regular daily smoker in 70.1% of the 1126 houses. With regard to area of residence, this was 67.7% inrural areas and 71.6% in urban areas (*p *> 0.05).

There was a daily or occasional smoker in 79% of homes where pregnant women lived and in 74% of homes where there was a child less than five years old.

Questions about tobacco smoking and related factors were asked to 2166 women and 1906 men aged 15 and over. The percentage of current smokers was found to be 29.5%. This rate was 11.9% in women and 49.6% in men (*p *< 0.001). The rate of ex-smokers was 2.1% in women and 9.1% in men (Table 1).

The percentage of current smokers was found to be higher in urban areas (34.5%) than rural areas (22.8%) (*p *< 0.01) and this was true for both sexes. In men the percentage was 53.6 % in urban and 43.7 % in rural areas (*p *< 0.001) and in women 16.6 % in urban and 5.8 % in rural areas (*p *< 0.001) (Table 1).

Cigarette consumption was expressed as the number of packs smoked/year. The mean consumption was found to be 6.5 packs/year in women and 17.9 packs/year in men. Mean total cigarette consumption by men was significantly higher than that of women (*p *< 0.001). Men living in rural areas consumed 20.1 packs/year, a greater amount than men living in urban areas (17.1 package/year) (*p *< 0.05). In spite of this, a significant difference was not found in cigarette consumption by women according to their area of inhabitance. (rural = 5.9, and urban = 6.7 packs/year) (*p *> 0.05).

Various characteristics of smokers are shown in Table 2. The percentage of current smokers was highest in the 30–34 age group in women (17.7%) and the 35–39 age group in men (67.3%). In women, the percentage of current smokers was also significantly higher in the 30–34 and 20–24 age groups and lower in the 50+ age group (*p *< 0.001). In men, it was found to be lower in the 15–19 age group (28.9%) than in the others (*p *< 0.001).

Current smoking percentages increased with the education level in both sexes. A peak was seen in secondary school and higher educated women (24.3%) (*p *< 0.001). In men, this was seen in those educated to high school level and above (54.3%),(*p *= 0.012).

Marital status was evaluated. Whilst marital status did not affect smoking in women, the percentage of current smokers was higher in married men (55.9%) than in those who were single or divorced (*p *< 0.0001) (Table 2).

2079 of women and 296 of men were unemployed. Although the employment status of women did not affect smoking (*p *= 0.62), the rate of current smokers amongst employed men was significantly higher than in unemployed men (*p *< 0.001), (Table 2).

In men, smoking behaviour differed with ethnicity. The rate of current smokers was higher in Turkish and Kurdish men than in Arabic and Zaza men (*p *= 0.02). Ethnicity did not affect smoking behaviour in women (*p *= 0.80) (Table 2).

Variables which were found statistically significant in the above bivariate analyses (age, gender, education, ethnicity, type of residence, marital and employment status), were included in a logistic regression model. Education level together with type of residence and, more strongly, age and gender were found to be associated with smoking after control of all other variables in the model (Table 3). The risk of smoking was 6.7 times higher in men than in women after the other variables were controlled. It was 3.8 times higher in the 35–39 age group than in the 15–19 age group. Smoking risk also increased with education levels and was higher in urban regions.

To evaluate the effects of smoking on the respiratory tract, the men and women who took part in this study were also asked if they had a cough of more than 15 days duration and whether or not sputum was present. The presence of a cough of more than 15 days duration was 8.7% in non-smokers and 15.8% in current smokers (*p *< 0.001). The presence of sputum was 8.3% in non-smokers and 14.7% in current smokers (*p *< 0.001) (Table 4). The presence of a cough of more than 15 days was 12.1% in ex-smokers and the presence of sputum was 9.9%.

## Discussion

Smoking is decreasing in developed countries [13-15], but increasing in developing countries including Turkey [1,2,16-18].

One of the targets of the WHO 'Health for All in the 21^st ^Century' policy is to reduce the proportion of smokers to lower than 20% in over 15s and to 0% in under 15s (Target 12) [3]. The rate was found to be 29.5% in over 15s in our study and this shows how far we are from this target. Also, passive smoking was present in 70% of the houses. These rates show the extent and importance of the problem in the region.

Smoking rates vary between the different regions of our country, as found in various studies [19-21]. In a study of the rural areas of Izmir, in the south-west of the country, the rate of current smokers was 13.2% in women and 64.3% in men aged 20 years and over [19]. In adolescents in Kocaeli, in the north-west, this rate was found to be 50.3% [20] compared to 32.1% for the same age group in our study. In another study in Aydın, again in the south-west, the rate was found to be 48.3% [21]. A study performed in 1988 showed that 62.8% of men and 24.3% of women aged 15 years and over were smoking in Turkey. The smoking rate of the total population was 43.6% [22]. These rates are higher than in our study. All this data shows that smoking is not only a big problem in the SEAP region but also in the rest of the country. However, it is pleasing that the rate of smoking in women in the SEAP region is approximately half that of the country.

Previous data on the prevalence of smoking in the South-east Anatolian Project (SEAP) Region is lacking. Due to this limitation, smoking trends in the region are not obvious. However the Ministry of Health reported it to be 29% for the whole south-east Anatolian region in 1993 [23] and this result is very close to the prevalence found in this study. Another study conducted in Elazig (a city near the SEAP region) in 1997, found the smoking prevalence to be 13.4% amongst females and 52.9% amongst males [24]. These findings indicate that the prevalence of smoking in the region has not increased with time. It is also reported that the previously increasing rate of cigarette consumption in Turkey fell in recent years [9].

In this study, factors associated with smoking were examined. Gender, age, type of residence and education level were determined as variables affecting smoking by logistic regression. Employment, marital status and ethnicity have also been found as variables associated with smoking but these associations did not remain significant after other factors were controlled.

Gender has been determined as the main variable affecting smoking by logistic regression. The smoking rate was 6.7 times higher in men than in women when the other variables were fixed. Cigarette consumption was also 3 times higher in men (the mean total was 18.0 packs/year in men, 6.5 packs/year in women). In a study performed in rural Izmir this mean was found to be 16.4 packs/year in men [19] compared with 20.1 packs/year in men in the rural SEAP region in our study. The result of our study is higher. Although a fewer number of men in rural areas of the region smoke than urban men, their total consumption of cigarettes is much higher. In summary, these results show us that smoking is a serious problem amongst men in the SEAP region and smoking-cessation campaigns should therefore target men here. However, it has been shown that south-eastern European regions are in stage III of the smoking epidemic and thus in the next few decades we expect an increase in smoking in women and a decrease in men [25].

Age was another factor affecting smoking. When the other variables were fixed, smoking was seen 3.8 times more in the 35–39 age group than in the 15–19 age group. Smoking rates were 7.8% in women and 28.8% in men in the 15–19 age groups. This indicates that men, especially, start smoking earlier. A significant increase in the level of current smokers was determined in the study in both men and women of the 20–24 age group. This suggests that the 15–19 and the 20–24 age groups are important targets for anti-smoking campaigns. These results show similarities with the results from 1993 [23].

It was important that higher smoking rates were associated with advancing age. However, the rate of smoking was seen to be decreasing in women over 45 years of age and in men over 50. One of the reasons for this decrease is thought to be due to people giving up smoking at this age due to smoking related health problems. The level of cessation of smoking was 2.5 times higher in this age group (10.1%) than younger ages (4.3%).

In this study, the smoking rate was seen to be 1.7 times higher in urban regions than rural regions. This was the case in both sexes (*p *< 0.001) and especially in women, with three times more urban women smoking than rural women. This indicates that urban women are a suitable target for future smoking-cessation campaigns. A higher level of education and economic independence can be speculated to be the contributing factors to this observation. Higher socio-economic levels are obtained by education. Smoking rates increased with education attainment in women. The highest smoking rates were in women who had graduated from secondary school or higher. Smoking was seen to be 1.7 times more prevalent in this group than in those who were classed as illiterate. Similar results were obtained regarding men. Higher education levels should be expected to reduce smoking rates. In a study in Estonia, a negative relationship between educational level and smoking has been determined [26]. A different result in our study is worrying for the effectiveness of education in Turkey. In several other studies conducted in Turkey, smoking was found to be highest in the well-educated groups in agreement with our study. [19,21,22].

Another result of this investigation was the high percentage of respiratory tract complaints in current smokers. The 'presence of a cough of more than 15 days duration' and the 'presence of sputum' was examined and these complaints were found significantly higher in smokers. The rate of 'presence of a cough of more than 15 days duration' was 8.7% in non-smokers and 15.8% in current smokers. The rate of 'presence of sputum' was 8.3% in non-smokers and 14.7% in current smokers (*p *< 0.001). These results are evaluated as an indicator of the negative effects of smoking on the respiratory system. It is thought that the higher rate of these complaints in men is due to them having a total cigarette consumption rate of three times higher than in women. Similarly, Steyn et al.(2002) reported that a dose-response was observed between the amount smoked and the presence of respiratory disease[18].

Tobacco smoke includes a lot of agents that cause lung cancer and chronic obstructive lung diseases and also exacerbates asthma [3,6,8]. Tobacco smoke also decreases birth weight and increases sudden infant death and allergies [3,4]. It affects not only the smokers themselves but also others around or living with them. This effect has been shown in many studies [4-8,27]. Continuous smoke exposure (passive smoking, involuntary smoking) affects the health of a household negatively, especially that of children and pregnant women. It has been found that passive smoking reduced the birth weight of infants of non-smoking mothers [4]. Passive smoking is accepted as one of the important factors in the frequency of respiratory tract infections in children. Therefore, the presence of passive smoking in the household was examined in this study. There was a regular daily smoker in approximately two-thirds of the houses. It would normally be expected that smoking rates would decrease in houses where children or pregnant women were living. However it is both important and worrying that our study showed this not to be the case in the households we examined, with smoking being present in two-thirds of houses where children or pregnant women lived.

The effect of passive smoking on respiratory tract complaints was also examined in this study. The percentage of respiratory tract complaints in non-smokers was similar whether or not they experienced passive smoking within the household. However, it would normally be expected that non-smokers experiencing passive smoking within the household would have a high percentage of respiratory tract complaints. It is likely that persons are not only exposed in their home, but also in cars, public transport and other community settings. Such exposure may preclude the ability to determine health effects due to the limited variance in the exposure variable. It can be speculated that the duration of exposure must also be evaluated in order to show the difference; perhaps long-term exposure is important in passive smoking.

## Conclusion

Active and passive smoking is a serious problem in the SEAP region. Exposure to passive smoking must be prevented with appropriate education programmes (especially in houses where children or pregnant women live).

## Competing interests

This project was supported by the SEAP Regional Development Management Ministry of the Republic of Turkey.

## Authors' contributions

AİB: design, data interpretation, manuscript writing, SS: design, data collection, data analyses, BÖ: design, data collection, data analyses, SÖ: design, data interpretation, manuscript writing, TŞ: data collection, data interpretation, HA: data collection, data analyses, GS: design, data collection, data interpretation, AC: design, data collection, data interpretation, YP: data collection, data interpretation, Eİ: design, data interpretation, FA: design, data interpretation

## Pre-publication history

The pre-publication history for this paper can be accessed here:



## Figures and Tables

**Table 1:** Smoking Behaviour amongst Men and Women Aged 15 Years and Over

**Type of Residence**	**Smoking Status**	**Men**		**Women**		**Total**	
		
		**n**	**%**	**n**	**%**	**n**	**%**
**Rural**	Current smoker	336	**43.7**	55	**5.8**	391	**22.8**
	Ex-smoker	85	11.0	13	1.4	98	5.7
	Non-smoker	348	45.3	879	92.8	1227	71.5

**Urban**	Current smoker	610	**53.6**	202	**16.6**	812	**34.5**
	Ex-smoker	88	7.7	33	2.7	121	5.1
	Non-smoker	439	38.6	984	80.7	1423	60.4

**Total**	Current smoker	946	**49.6**	257	**11.9**	1203	**29.5**
	Ex-smoker	173	9.1	46	2.1	219	5.4
	Non-smoker	787	41.3	1863	86.0	2650	65.1

	**Total**	1903	100.0	2166	100.0	4072	100.0

Statistical differences in percentages of current smokers between rural and urban *p *< 0.01.

Statistical differences in percentages of current smokers between men and women *p *< 0.0001.

Statistical differences in percentages of current smokers between rural and urban areas in women (*p *< 0.000).

Statistical differences in percentages of current smokers between rural and urban areas in men (*p *< 0.000).

**Table 2:** The Distribution of Various Characteristics and Current Smoking amongst Men and Women Aged 15 Years and Over

**Characteristics**		**Women**			**Men**		
		
		**Total**	**Current smoker**		**Total**	**Current smoker**	
		**N**	**n**	**%**	**N**	**n**	**%**
Age groups (year)	15–19	500	39	7.80	398	115	28.89
	20–24	368	62	16.85	267	142	53.18
	25–29	277	39	14.08	228	132	57.89
	30–34	175	31	17.71	176	107	60.80
	35–39	219	28	12.79	199	134	67.34
	40–44	137	20	14.60	142	82	57.75
	45–49	135	11	8.15	118	70	59.32
	50+	358	27	7.54	375	164	43.73

	Statistical results	*x*^2 ^= 37.06	*P *< 0.001		*x*^2 ^= 124.32	*P *< 0.001	

**Education**	Illiteracy	1130	106	9.38	264	119	45.08
	Literacy	208	23	11.05	221	103	46.50
	Graduated from primary school	635	81	12.75	927	461	49.73
	Graduated from secondary school	77	20	25.97	192	101	52.60
	Graduated from high school and higher	116	27	23.27	298	162	54.36

	Statistical results	*x*^2 ^= 36	*P *< 0.001		*x*^2^(*for linear trend*) = 6.2 *p *= 0.012		

**Marital status**	Married	1309	170	12.99	1245	696	55.90
	Single	703	70	9.96	645	247	38.29
	Widowed/Divorced	160	17	10.63	13	3	23.08

	Statistical results	*x*^2 ^= 4.26	*p *= 0.118		*x*^2 ^= 56.41	*P *< 0.001	

**Employment**	Employed	93	9	9.68	1606	824	51.31
	Unemployed	2079	248	11.93	296	121	40.88

	Statistical results	*x*^2 ^= 0.24	*p *= 0.62		*x*^2 ^= 10.46	*p *= 0.0012	

**Ethnicity**	Turkish	731	91	12.45	691	351	50.80
	Kurdish	1223	144	11.77	1034	526	50.87
	Arabic	150	16	10.67	141	56	39.72
	Zaza	67	6	8.96	37	13	35.14

	Statistical results	*x*^2 ^= 0.99	*p *= 0.802		*x*^2 ^= 9.66	*p *= 0.022	

**Table 3:** The results of logistic regression *

**Independent**	**Variables**	**Smoking**		
		
		***p***	**Odds Ratio**	**Confidence Interval (95%)**
**Gender**	Women		1	1
	Men	*<0.001*	6.76	5.63–8.12
**Age**	15–19		1	1
	20–24	*<0.001*	2.86	2.19–3.74
	25–29	*<0.001*	2.89	2.18–3.83
	30–34	*<0.001*	3.48	2.56–4.74
	35–39	*<0.001*	3.78	2.82–5.08
	40–44	*<0.001*	3.12	2.22–4.38
	45–49	*<0.001*	2.81	1.96–4.02
	50+	*<0.001*	2.06	1.56–2.73
**Type of residence**	Rural		1	1
	Urban	*<0.001*	1.74	1.48–2.04
**Education**	Illiteracy		1	1
	Literacy	*<0.05*	1.35	1.01–1.80
	Graduated from primary school	*<0.01*	1.43	1.14–1.79
	Graduated from secondary school	*<0.001*	1.76	1.26–2.46
	Graduated from high school and higher	*<0.001*	1.64	1.22–2.20
**Employment**	Unemployed		1	1
	Employed	*>0.05*	1.14	0.93–1.40
**Ethnicity**	Arabic + Zaza		1	1
	Turkish	*>0.05*	1.04	0.77–1.41
	Kurdish	*>0.05*	1.15	0.86–1.53
**Marital status**	Single		1	1
	Married + widowed/divorced	*>0.05*	1.30	0.99–1.71

*Gender, age, education, ethnicity, type of residence, marital and employment status were included in logistic regression analysis as covariates.

**Table 4:** Presence of Cough and Sputum in Non-Smokers, Ex-Smokers and Current Smokers

	**Cough Of More Than 15 Days**				**Sputum**			
	
	**Yes**		**No**		**Yes**		**No**	
	
**Smoking status**	**n**	**%**	**n**	**%**	**n**	**%**	**n**	**%**
Non-smoker	190	**8.7**	1993	**91.3**	182	**8.3**	1998	**91.7**
Ex-smoker	23	**12.1**	167	**87.9**	20	**9.9**	182	**90.1**
Current smoker	163	**15.8**	851	**84.2**	149	**14.7**	865	**85.3**
Statistical results	*x*^2 ^= 38.3		*p *< 0.0001		*x*^2 ^= 30		*p *< 0.0001	
